# Factors Affecting the Abundance of Leaf-Litter Arthropods in Unburned and Thrice-Burned Seasonally-Dry Amazonian Forests

**DOI:** 10.1371/journal.pone.0012877

**Published:** 2010-09-21

**Authors:** Juliana M. Silveira, Jos Barlow, Julio Louzada, Paulo Moutinho

**Affiliations:** 1 Ecology Department, Universidade Federal de Lavras-UFLA, Lavras, Minas Gerais, Brazil; 2 Zoology Department, Museu Paraense Emílio Goeldi-MPEG, Belém, Pará, Brazil; 3 Lancaster Environment Centre, Lancaster University, Lancaster, United Kingdom; 4 Instituto de Pesquisa Ambiental da Amazônia-IPAM, Brasília, Brazil; University of Zurich, Switzerland

## Abstract

Fire is frequently used as a land management tool for cattle ranching and annual crops in the Amazon. However, these maintenance fires often escape into surrounding forests, with potentially severe impacts for forest biodiversity. We examined the effect of experimental fires on leaf-litter arthropod abundance in a seasonally-dry forest in the Brazilian Amazon. The study plots (50 ha each) included a thrice-burned forest and an unburned control forest. Pitfall-trap samples were collected at 160 randomly selected points in both plots, with sampling stratified across four intra-annual replicates across the dry and wet seasons, corresponding to 6, 8, 10 and 12 months after the most recent fire. Arthropods were identified to the level of order (separating Formicidae). In order to better understand the processes that determine arthropod abundance in thrice-burned forests, we measured canopy openness, understory density and litter depth. All arthropod *taxa* were significantly affected by fire and season. In addition, the interactions between burn treatment and season were highly significant for all *taxa* but Isoptera. The burned plot was characterized by a more open canopy, lower understory density and shallower litter depth. Hierarchical partitioning revealed that canopy openness was the most important factor explaining arthropod order abundances in the thrice-burned plot, whereas all three environmental variables were significant in the unburned control plot. These results reveal the marked impact of recurrent wildfires and seasonality on litter arthropods in this transitional forest, and demonstrate the overwhelming importance of canopy-openness in driving post-fire arthropod abundance.

## Introduction

Fire is frequently used as a tool to manage land for cattle ranching and annual crops in the Amazon [1]. However, a combination of factors, including forest degradation, logging, and abnormal drought events linked to global climate change, mean that these maintenance fires often escape into surrounding forests [1]–[4]. These fires have been identified as one of the major factors regarding the potential for climate-induced dieback of the Amazon forest [5].

Scientific understanding of the causes and consequences of these Amazonian wildfires has grown since they were first highlighted as a major issue over 20 years ago [2]. It is now known that wildfires induce microclimatic changes in burned forests and neighboring areas, increasing temperature, reducing forest moisture and consequently enhancing the susceptibility to recurrent fires [6]–[8]. Fire also induces changes in plant composition and soil properties [9]–[10], respectively, and leads to a substantial loss of above-ground biomass [7] contributing to greenhouse gas emission [11]. It is also possible that fire leads to a significant impoverishment of faunal biodiversity, although to date most studies have focused on vertebrates [12].

The effects of fire on surface-active litter arthropods are relatively well studied in some fire-prone ecosystems where fire is a natural and frequent event including sclerophyllous Eucalyptus forests [13]–[14], tropical savannas [15]–[16], conifer forests [17]–[18], and grasslands [19]. However, our knowledge in humid tropical forests is very poor and is restricted to just two short-term studies in Amazon forests [20]–[21]. The abundance of some arthropods can be related to the amount of leaf-litter in a burned neotropical savanna [22] but the processes that drive changes in arthropod communities in burned humid forests have not been examined, although it seems likely that changes in forest structure such as the enlargement of canopy gaps, regeneration, and the combustion of leaf-litter could influence arthropod communities.

The objective of this study was to examine the effects of recurrent fires on the leaf-litter arthropod *taxa*, using an Amazonian transitional forest that had undergone experimental recurrent burns (see Experimental fire treatments in Material and Methods section). Since these forests have a marked dry season [23] the single and combined effects of seasonality and fire treatment (burned and unburned) were also investigated. It was hypothesized that (1) recurrent fires decrease the abundance of arthropods, (2) there is a strong influence of seasonality in these transitional forests, (3) there is an interaction between disturbance treatment and seasonality [24] and (4) different environmental variables determine abundance of arthropods in the unburned and thrice-burned forest.

## Results

### Forest structure

Recurrent fires caused marked and consistent changes in forest structure. Forest canopy was greater than three times more open in the thrice-burned forest, while both understory density and litter depth were significantly reduced in thrice-burned forest (Table 1). There was no significant difference in canopy openness across seasons. Although understory density and litter depth were significantly different across seasons, the changes in the mean values were minimal, and most of the variance was explained by burn treatment (Table 1).

**Table 1:** Differences (mean ± SE) between forest structure variables collected in unburned and thrice-burned plots.

		Canopy openness (%)	Understory density (un)	Litter depth (cm)
**Unburned (** ***n*** ** = 162)**	February	6.54±0.67	3.11±0.12	4.43±0.15
	August	6.55±0.68	3.33±0.15	4.02±0.13
**Thrice-burned (** ***n*** ** = 162)**	February	22.07±1.60	0.86±0.12	1.35±0.06
	August	22.15±1.59	0.69±0.08	1.01±0.02
**Analysis of Deviance**	Month	<0.001	<0.001	<0.001
	Treatment	<0.001	<0.001	<0.001
	Month x Treatment	= 0.001	>0.05	= 0.01

Statistical results are from Analysis of Deviance using chi-square to test significance and based on quasi-Poisson (understory density and litter depth), and quasibinomial errors (proportion of canopy openness).

### Leaf-litter arthropod

We collected a total of 25,440 leaf litter arthropods in the unburned control plot and 40,853 in the thrice-burned plot. These were distributed across 26 groups (25 orders and the family Formicidae). We analyzed data for all arthropods together and for the most abundant groups (*taxa* where we caught more individuals than the total number of pitfall traps used- i.e. only those with >160 individuals overall, Table S1). There was no correlation between spatial location within plots and community structure in any seasonal replicate (P-values ranged between 0.19 and 0.98 for the eight RELATE tests, based on all arthropods).

**Figure 1:**
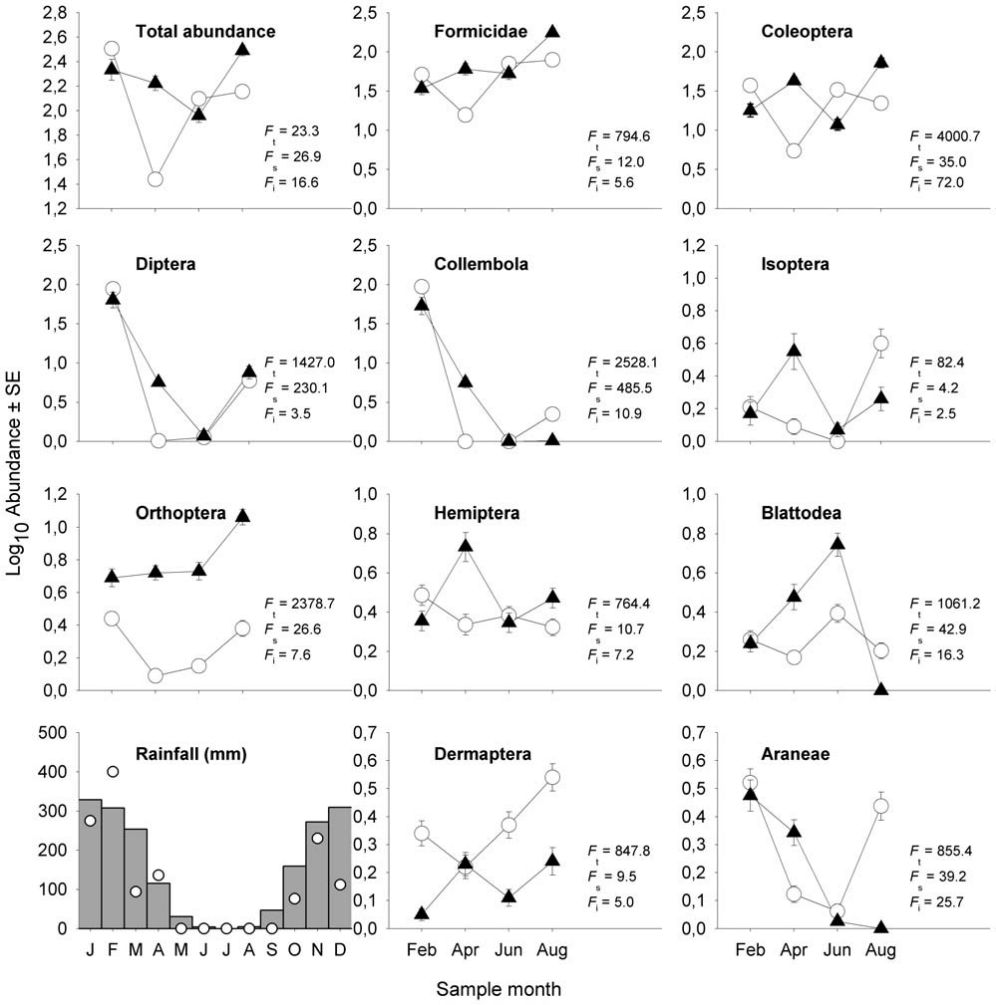
Mean (± SE) abundance for all arthropods and the ten most abundant orders across the four sample periods, and rainfall (28 year average monthly) across the year. Circles represent the unburned plot and triangles represent the thrice-burned plot. Circles in rainfall panel represent rainfall for the year of the study. Months in the *x* axis represent 6, 8, 10 and 12 months post-fire, respectively. *F*-values for Analysis of Deviance tests for treatment, sample and interaction between both are shown in this order in the panels. For all tests degrees of freedom for treatment  = 1, 302, for sample  = 3, 299, for plot x sample  = 3, 296. All tests for treatment were highly significant (*p*<0.001). All tests for sample season were significant at *p*<0.001, except for Isoptera (*p* = 0.005). The interaction between treatment and season was significant for all *taxa,* except for Isoptera (*p* = 0.06).

The effects of burn treatment and seasonal replicate were highly significant for all arthropods together, and for almost all orders (Figure 1). Within each arthropod *taxa*, treatment (whether a plot was burned or not) explained more of the variance than the season when sampling was conducted (see *F*-values in Figure 1). The interactions between burn treatment and seasonal replicate were almost all highly significant (Figure 1). The changes in the Formicidae and Coleoptera were particularly pronounced over time, as these *taxa* showed an opposite pattern of abundance in the burned and control treatments. Orthoptera was the only *taxon* where abundance was consistently higher in the thrice-burned plot across all seasonal replicates. The other orders had distinct responses to burn treatment and seasonality (or time since fire).

### Relating leaf-litter arthropod abundance with forest structure

**Figure 2:**
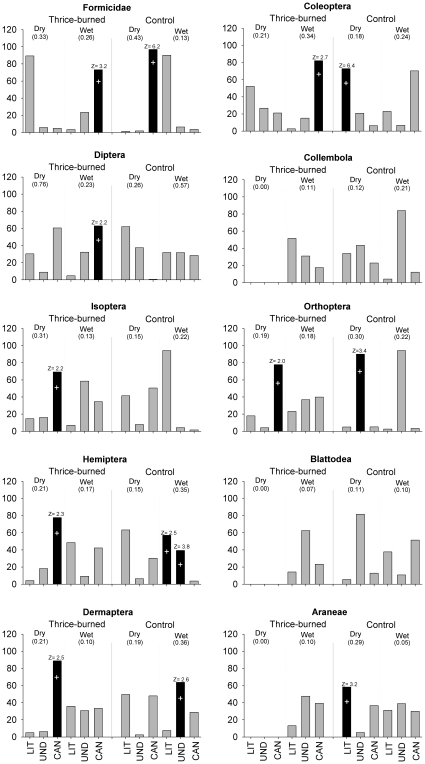
Distribution of the percentage of independent effects of environmental variables on arthropods in thrice-burned and unburned plots, and in the dry and wet seasons. Black bars represent significant effects (*p*<0.05) as determined by randomization tests. Z-scores for the generated distribution of randomized I's (I value  =  the independent contribution towards explained variance in a multivariate dataset) and an indication of statistical significance. Z-scores are calculated as (observed – mean (randomizations))/SD(randomizations), and statistical significance is based on upper 0.95 confidence limit (Z≥1∶65). Positive or negative relationships are shown by + or –, respectively. *R* ^2^_dev_ (displayed in parenthesis under the seasons) is the total deviance explained by a generalized linear model including the three measured variables. LIT  =  litter depth, UND  =  understory density and CAN  =  canopy openness.

Within the thrice-burned plot, seven out of 20 randomization tests revealed a significant influence of canopy openness on arthropods (Figure 2), but none of the other forest structure variables were significant. This contrasts with the unburned forest, where six of the 20 tests were significant, but all three environmental variables were significant at some point (leaf-litter for Coleoptera and Araneae in the dry season and for Hemiptera in the wet season; understory density for Orthoptera in the dry season, and for Hemiptera and Dermaptera in the wet season; and canopy openness for Formicidae in the dry season). None of the three forest structure variables predicted the abundance of Blattodea and Collembola in either treatment or season (Figure 2).

## Discussion

This is the first study that has examined the likely impact of recurrent fires on leaf-litter arthropods abundance and activity in tropical forests. Our hypotheses that recurrent fires reduced the total abundance of arthropods was rejected, but our three other hypotheses were strongly supported: seasonality had a strong effect on arthropods abundance, there was a strong interaction between burn-treatment and our seasonal replicates (Figure 1), and different aspects of forest structure influenced arthropod abundance in unburned and thrice-burned forests (Figure 2). Although our results were from one burned and one unburned 50 ha site, large plot size and the lack of spatial autocorrelation within plots suggests this did not influence our results.

The complex changes in abundance revealed in our study (Figure 1) make it very difficult to attribute biologically meaningful explanations for the patterns we found. Some of the complexity may reflect the large number of different species contained within each *taxa* [25]. This is further complicated by the paucity of comparable data from other tropical forests, and there are methodological differences with the two previous studies that do exist. Although [20] also conducted seasonal replicates, their study was conducted four years after the fire event, it was based on much larger pitfalls designed to catch herpetofauna and the forests they examined were both burned and logged. [21] focused on once-burned forest one-year after fire in a Brazilian humid tropical forest, and they did not conduct seasonal replicates.

### Effects of recurrent fires on forest structure

As in previous studies [26], recurrent fires severely affected the forest structure, increasing canopy openness, and reducing understory density and litter depth. It is likely that the increased canopy openness exerted a strong influence on forest microclimate. For example, temperature is 3–4°C higher in canopy gaps [27] and a sufficient increase in gap density can also increase the temperature of the forest as a whole [6], [28]–[29]. Although canopy gaps also favor the rapid regeneration of shrubs, lianas and large herbs [30], this regeneration had not had time to develop since the last fire event in our study plot (Table 1). Furthermore, the reduced litter depth in the burned treatment can be linked to the time since last fire, which was insufficient to accumulate leaf-litter. Although changes in litter quality could influence the arthropod community [22], [31] we did not address this in our study.

### Relationships between forest structure and leaf-litter arthropod

Our results suggest that canopy openness is the most important factor affecting the arthropod fauna in burned forests, presumably because the canopy gaps created by recurrent fires (as a result of tree mortality) exert such a strong effect on temperature and humidity (see above) that they overwhelm the importance of other more subtle environmental variables that can influence the arthropod community in unburned forests (Figure 2). This result may not be restricted to surface-active arthropod. For example, microarthropods are also closely related to leaf-litter humidity [32], and canopy openness was the best single predictor of understory bird communities in once and twice-burned Amazonian forests [33]. Furthermore, canopy openness is an important determinant of animal communities following other forms of forest degradation, including selective logging or even edge effects in forest fragments [6], [34].

Canopy openness is also strongly linked to the density of plants and young leaves in the understory, increasing the food resource for herbivorous arthropods, which in turn can increase resources for arthropods predators [35]. However, this mechanism is unlikely to explain the higher arthropod abundance in the thrice-burned plot when compared to the control plot, as the understory density was actually lower in the thrice-burned plot (probably because the understory vegetation had not yet had time to recover within one year of the most recent fire). Instead, the high abundance of arthropods such as the herbivorous Orthoptera in the thrice-burned plot can be related to the leaf quality for herbivorous, as young leaves and pioneers species generally have fewer defenses against herbivores [36].

Although the patterns we present here clearly demonstrate the importance of recurrent fires in structuring arthropod communities in seasonally dry tropical forests, the results should be interpreted in a general sense as specimens were only identified to a very coarse taxonomic level, and changes in one single species in each group could bias the results [25]. For example, the results for the Formicidae were certainly influenced by the increasing occurrence of the leaf-cutting ant *Atta* in the burned plot, and are unlikely to provide an accurate reflection of the full complexity of changes within the ant fauna. In addition, while pitfall traps are appropriate for samplings some groups (e.g. ants and ground beetles) they are less effective for collecting groups such as grasshoppers and termites that occupy a diverse range of microhabitats extending well beyond the leaf-litter. However, as highly significant effects were observed at the crudest of taxonomic resolutions, we can only surmise that a more detailed investigation would reveal an even greater influence of recurrent fires, including a high degree of community turnover and species extirpations in areas that have succumbed to recurrent burns.

Finally, many of these orders play an important role in regulating ecosystem functions such as decomposition [37], seed removal and dispersal [38], and soil bioturbation [39]. We can therefore presume that the changes in abundance and composition of arthropods following recurrent burns that we demonstrate here will have significant cascading consequences for ecosystem functioning, and are a priority area for further investigation.

Although fire intensity may be lower during a third annual burn in this transitional Amazon forest [23], the sequence of repeated fire disturbance has a strong impact upon leaf-litter faunal communities. In contrast to our predictions, recurrent fires increased the total number of arthropods we sampled. However, these patterns were complex, and were highly *taxon* and season dependent. Moreover, the interaction between fire and seasonality can obscure or confuse the influence of fire disturbance. This has important consequences for short-term studies in seasonal forests [40] which may not provide robust or representative results. Of the changes in forest structure that were observed in thrice-burned forest, the increase in the number of canopy gaps appears to be the most important, presumably because a very open canopy alters the microclimate with cascading effects on the leaf-litter dwelling arthropods. Our understanding of the mechanisms that drive these changes would be improved through more detailed work on individual species or functional groups [25], as well as by experimental manipulations of abiotic conditions on arthropod feeding behavior and reproductive success.

## Materials and Methods

### Study area

The study was carried out in a transitional Amazonian forest in the municipality of Querência in the state of Mato Grosso, Brazil (Fazenda Tanguro; 13°04′35.39″S, 52°23′08.85″W), 30 km north of the Amazonia-*cerrado* (Brazilian savannah) boundaries. The site (150 ha) was established in the property's legally protected forest reserves and was surrounded by more than 1 km of native forest on three sides, while one side was adjacent to pasture [23]. The 150 ha site was divided in three 50 ha plots adjacent to each other: unburned control forest, annually burned forest (experimentally burned in 2004, 2005 and 2006), tri-annually burned forest (experimentally burned in 2004). Vegetation and soil type were sampled before the experiments of fire took place and were similar between the three plots [23]. Average annual rainfall is around 1500 mm and average temperature around 26°C. There is a marked dry season from May to September (Figure 1, [23]). This study was carried out from February - August 2007 (before the 2007 fires, see next section), 6–12 months after the last fire. Sampling was conducted in the unburned and thrice-burned forest plots that were separated by the once-burned plot. We selected the thrice-burned plot because we were interested in the effects of extreme forest disturbance, but recognize that forests rarely burn annually in Amazon.

### Experimental fires treatments

This project is part of a larger experiment which aims to identify and quantify the variables that control the fire behavior in transitional forests. The larger experiment has been developed in the 150 ha site. All experimental burns took place at the end of the dry season (August–September). Fires were set using kerosene drip torches along the N-S trails (as kerosene was only used along narrow lines 50 m apart, we do not think this treatment would affect the leaf-litter arthropods we sampled). A total of 10 km of firelines were set per plot during 3–4 consecutive days from 9h00 to 16h00. These fires often smouldered or went out during the more humid nights, and were therefore relit the following day. Fires typically burned leaf litter and fallen twigs and branches, but not the standing trees [23].

### Spatial scale and experimental design replication

Significant logistical, legal and financial constraints meant the experimental design lacked replication at the plot level. However, we believe that this limitation does not undermine the contribution these data make for understanding the effects of fire on leaf-litter arthropods in tropical forests. Importantly, the close proximity of the plots and pre-burn data indicate that the plots were similar in forest structure and composition before burning began [23]. Furthermore, the lack of replication was compensated by the large size of each study plot (50 ha): in contrast, many previous studies in different biomes may have underestimated the effects of large-scale fires (such as those that occur in tropical forests) by sampling in small burned plots (0.25–1 ha) that can be rapidly colonized by arthropods [14–17, 41–45], or may be affected by the surrounding unburned vegetation. The study also had a very high level of spatial and temporal replication within plots, with environmental measurements taken at an appropriate scale for leaf-litter arthropods. In addition, we tested the spatial independence of our within-plot replication (see below).

### Forest structure

Trails were placed out in N-S directions and marked every 50 m in E-W directions in the two plots, forming a grid where the forest structure and the arthropods sampling were undertaken. Litter depth, understory vegetation density and canopy openness were recorded in 3×3 m quadrats placed 2 m from the trail at every grid point (*n* = 324 for each plot) in February (wet season) and August (dry season). Litter depth was measured with a ruler at the four corners of each quadrat. Understory density was indexed by counting how many times live vegetation touched a 2.5 m high vertical pole, placed at the four corners of each quadrat. Canopy openness was estimated using digital hemispherical photographs taken in the centre of each quadrat and analyzed using Gap Light Analyzer [46]. All sample points (*n* = 324) were used to characterize the environmental conditions in each forest treatment, while a subset of these were used to characterize the grid points where arthropods were collected (*n* = 160) in each treatment, divided equally across the two seasons), allowing us to examine the relationship between forest structure and arthropods abundance.

### Leaf-litter arthropod sampling

Arthropods were sampled using pitfall traps which estimate the relative abundance of surface-active arthropods, thereby providing a measure of their importance on the forest floor [47]. Each trap consisted of a 750 ml plastic cup (12 cm surface diameter) and a plastic cover to prevent rain overflow. These were half filled with 70% ethanol and a few drops of detergent, and this solution was replaced every other day. Edge effects were avoided by placing pitfall traps at least 100 m from the pasture and adjacent treatments. Arthropods were sampled at four times throughout the study period; in February and April representing the wet season, and in June and August, representing the dry season. During each sampling expedition, 40 points were randomly selected in each grid and one pitfall trap was placed 2 m from the trail, and left for seven days (*n* = 160 across all intra-annual replicates in each plot). Therefore we sampled 6, 8, 10 and 12 months after the last fire in the thrice-burned plot. These intra-annual replicates are referred to as seasonal replicates from hereon, although we are not able to separate the potential influence of time since the last fire with that of seasonality. Arthropods were preserved in 70% alcohol, identified to order level and counted. In addition, the family Formicidae was separated from the Hymenoptera. For all arthropod *taxa*, patterns of abundance rather than occurrence were analyzed, as these reflect activity and make our results comparable with two previous studies that conducted pitfall trapping in burned Amazonian forests.

### Analyses

Analysis of Deviance was used to assess the influence of burn treatment and seasonality on forest structure, the abundance of all arthropods, and the abundance of the ten most abundant orders. We used quasi-Poisson error structure for count data, and quasi-binomial errors in the case of canopy cover, which was proportion data [48]. These were conducted in the R statistical program [49].

Spatial autocorrelation within plots was assessed using Mantel-type RELATE tests in Primer 5.0. Similarity matrices were constructed based on normalized Euclidean distance between the locations of the sampling points. These were correlated with similarity matrices constructed based on arthropod composition using Bray-Curtis similarity. Eight correlations were undertaken in total (i.e. for each of the 2 treatments and 4 seasonal replicates).

Hierarchical partitioning [50] were used to compare the relative and independent importance of our three environmental variables (litter depth, understory density and canopy openness) on the abundance of the arthropod fauna. Because of the overwhelming importance of treatment and season on abundance, four separate analyses for each dependent variable were conducted, allowing us to examine whether arthropods are influenced by similar environmental factors in each season and burn treatment. These analyses were restricted to the ten most abundant *taxa*.

Hierarchical partitioning is a multiple-regression technique designed to identify - by using all possible model combinations - the variables that have the greatest independent influence on the dependent variable, providing a measure of the effect of each variable that is largely independent from that of other variables [50]–[51]. Models used quasi-Poisson errors, and we evaluated competing models based on the R^2^ goodness of fit statistic. The significance of independent effects was calculated using a randomization test with 1000 iterations [52]. Hierarchical partitioning and associated randomization tests were implemented using the hier.part package in the R statistical program [49].

## Supporting Information

Table S1 Mean arthropod abundance and standard error in unburned and thrice-burned forest plots in the four sampling periods.
(0.08 MB DOC)
