# Supplementary material for: Factors Affecting the Abundance of Leaf-Litter Arthropods in Unburned and Thrice-Burned Seasonally-Dry Amazonian Forests
Source: PLoS One. 2010 Sep 21;5(9):e12877. doi: 10.1371/journal.pone.0012877 (PMC2943466; doi:10.1371/journal.pone.0012877)
Supplement: Table S1 — Mean arthropod abundance and standard error in unburned and thrice-burned forest plots in the four sampling periods. (0.08 MB DOC) [file pone.0012877.s001.doc]

Table S1. Arthropod abundance in unburned and thrice-burned forest plots in the four sampling periods.

| **Arthropods group** | **Sample period** | **Control** | | **Burned** | |
| --- | --- | --- | --- | --- | --- |
|  |  | Mean | SE | Mean | SE |
| **Formicidae** | February | 58.69 | 5.68 | 51.16 | 7.11 |
|  | April | 21.03 | 3.24 | 142.31 | 58.90 |
|  | June | 91.58 | 11.11 | 99.87 | 24.37 |
|  | August | 115.95 | 18.09 | 244.48 | 34.97 |
| **Coleoptera** | February | 42.61 | 3.77 | 28.27 | 4.23 |
|  | April | 5.95 | 0.97 | 49.67 | 4.61 |
|  | June | 36.45 | 2.46 | 17.71 | 2.25 |
|  | August | 26.08 | 2.11 | 88.2 | 6.82 |
| **Diptera** | February | 117.92 | 14.99 | 125.43 | 22.69 |
|  | April | 0.03 | 0.03 | 5.53 | 0.58 |
|  | June | 0.28 | 0.17 | 0.37 | 0.17 |
|  | August | 6.32 | 0.69 | 12.33 | 2.25 |
| **Collembola** | February | 110.11 | 10.08 | 105.11 | 14.76 |
|  | April | 0 | - | 6.86 | 0.96 |
|  | June | 0 | - | 0 | - |
|  | August | 1.84 | 0.33 | 0.03 | 0.03 |
| **Isoptera** | February | 2.67 | 1.83 | 5.57 | 4.91 |
|  | April | 1.23 | 0.96 | 25.17 | 18.04 |
|  | June | 0 | - | 1.03 | 0.85 |
|  | August | 8.14 | 2.29 | 3.68 | 2.02 |
| **Orthoptera** | February | 2.22 | 0.31 | 5.22 | 0.65 |
|  | April | 0.36 | 0.13 | 5.19 | 0.65 |
|  | June | 0.70 | 0.22 | 6.58 | 0.73 |
|  | August | 2.00 | 0.33 | 12.50 | 1.07 |
| **Hemiptera** | February | 2.86 | 0.44 | 1.92 | 0.39 |
|  | April | 1.97 | 0.49 | 8.67 | 2.25 |
|  | June | 1.93 | 0.29 | 2.21 | 0.52 |
|  | August | 1.53 | 0.24 | 2.85 | 0.47 |
| **Blattodea** | February | 1.22 | 0.26 | 1.11 | 0.26 |
|  | April | 0.64 | 0.15 | 3.61 | 0.88 |
|  | June | 2.05 | 0.36 | 7.26 | 0.86 |
|  | August | 0.89 | 0.20 | 0 | - |
| **Dermaptera** | February | 1.67 | 0.33 | 0.19 | 0.08 |
|  | April | 1.03 | 0.24 | 1.03 | 0.24 |
|  | June | 1.93 | 0.32 | 0.53 | 1.18 |
|  | August | 3.29 | 0.44 | 1.33 | 0.34 |
| **Araneae** | February | 3.08 | 0.44 | 2.92 | 0.46 |
|  | April | 0.46 | 0.12 | 1.67 | 0.28 |
|  | June | 0.28 | 0.15 | 0.11 | 0.06 |
|  | August | 2.61 | 0.58 | 0 | - |
